# Supplementary figures and images for: Why are song lyrics becoming simpler? a time series analysis of lyrical complexity in six decades of American popular music
Source: PLoS One. 2021 Jan 13;16(1):e0244576. doi: 10.1371/journal.pone.0244576 (PMC7806124; doi:10.1371/journal.pone.0244576)

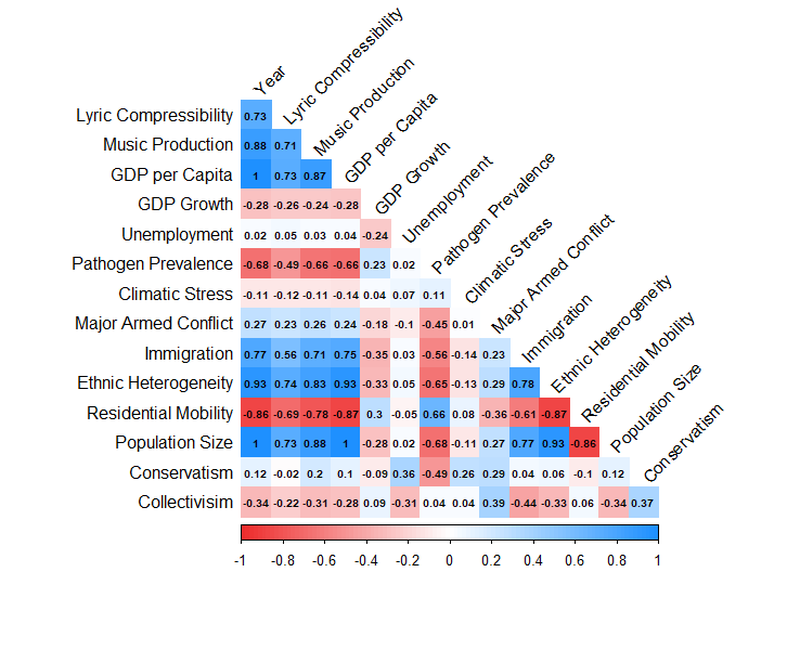

Supplement: S1 Fig — (TIF) [file pone.0244576.s001.tif]
