# Supplementary material for: Why are song lyrics becoming simpler? a time series analysis of lyrical complexity in six decades of American popular music
Source: PLoS One. 2021 Jan 13;16(1):e0244576. doi: 10.1371/journal.pone.0244576 (PMC7806124; doi:10.1371/journal.pone.0244576)
Supplement: S1 File — (DOCX) [file pone.0244576.s002.docx]

Why are songs becoming simpler? A time series analysis of lyrical complexity in six decades of American popular music

Michael E. W. Varnum, Jaimie Arona Krems, Colin Morris, Alexandra Wormley,

Igor Grossmann

**Lyrical Compressibility**

We sought out the lyrics for all songs that entered the Billboard Hot 100 from its inception (in 1958) until the end of 2016. Data collection took place in mid 2017 and some song were retrieved from 2017, however we included only data through 2016 in order to ensure comparability of the data across years. Partial 2017 data is available at (<https://osf.io/qnsmj/)> but was not used in any analyses reported in this paper. We used [billboard.py](https://github.com/guoguo12/billboard-charts) to access metadata for charting songs, and sought out the corresponding lyrics by visiting lyrics websites. Of the 27,404 songs that charted in this interval, we were able to glean quality lyrics for slightly more than half (*N* = 14,661).

We stored the lyrics of each song in an ASCII text file. A song's uncompressed size was simply the size of its text file in bytes, or, equivalently, its length in characters. We compressed each file using the software gzip (www.gzip.com) with the -9 option (maximum compression, at the cost of speed). Importantly, we do not consider the results of the full DEFLATE compression implemented by gzip, which consists of an LZ77 step followed by a Huffman coding step. The former exploits repeated substrings in the input text to produce a more compact representation. The latter exploits varying character frequencies, which is unlikely to bear on a listener's perception of repetitiveness or redundancy. Instead, we used the software infgen (<https://github.com/madler/infgen>) to parse the LZ77 structure from the compressed encoding produced by gzip, bypassing the Huffman coding step.

We counted text that was untouched by LZ77 in the normal way (1 byte per character). We counted each match (i.e. each reference to an earlier substring) as costing 3 bytes. This accords closely with the reality of typical implementations of LZ77 (in terms of number of bits used to encode match length and distance), but more importantly, it's appropriate to our data.

If we classify repeated strings as "true" repetitions (i.e., deliberate repetitions used to some poetic effect which listeners will perceive as such), and artefactual or incidental repetitions (e.g., repetitions of minor function words like "the" or "and" in unrelated contexts, or simply character ngrams that are common in English morphology, like "ing", or "er"), then we're faced with a trade-off between precision and recall, for which the cost of a match acts as a fulcrum. Smaller costs increase recall at the cost of precision and vice versa. 3 bytes is small enough to allow matches on some short content words, but these are relatively inefficient. A match of the shortest length (e.g., "then") turns a 4 byte string into 3 bytes, for a reduction of 1 byte or 25%. A match of length 6 (e.g., "lovers") gives a 50% reduction. These savings pale compare to the long repeated sections that abound in pop lyrics; for example, the chorus of "Call Me Maybe" which repeats 4 times and is compressed 98%.

**Compressibility of Successful Song Lyrics vs. All Song Lyrics**

It is worth noting that the measure of average lyrical compressibility was restricted to songs that were highly successful, those among that were among the Top 100 for sales and streaming during a given year. Patterns of average compressibility for this subset of songs may differ from those for *all* songs produced in a given year. To our knowledge, no such comprehensive database of all song lyrics organized by year of release exists, but it may become available in the future. Further, the primary focus of the present study was how the characteristics of successful cultural products may vary as a function of number of choices in the environment, rather than how *all* cultural products might vary in such conditions. Future research may explore whether the cross-temporal dynamics in the features of cultural products are systematically different among products that succeed vs. all such products.

**First Order Auto-Correlation and Tiohkin-Hruschka Corrected p-values.**

We computed a corrected p-values for the key correlations between our composite measure of novel songs produced per year and average lyrical compressibility to account for the fact that our time series were non-stationary using the Tiokhin-Hurschka method (*20*). The PCA based music production index of available novel songs had first order autocorrelation of .919 and the lyrical compressibility index had first order autocorrelation of .869. The Tiokhin-Hruschka method creates significance thresholds for correlations that are adjusted for the autocorrelation observed in the time series by generating a bootstrapped null-distrubtion for 10000 datasets containing the same amount of data points and the same degree of autocorrelation as observed in the actual time series. Thus, this method can be used to create *p*-values adjusted for the degree to which autocorrelation is present in time series. R code and resulting adjusted significance threshold are provided below:

###Compressibility & PCA-based Novel Music Production Composite###

simnum<-10000

simul <- matrix(nrow=simnum, ncol=1, 0)

for (i in 1:simnum){

ar.sim<-arima.sim(model=list(ar=c(.869)),n=59)

ar.sim2<-arima.sim(model=list(ar=c(.919)),n=59)

simul[i] <- cor(ar.sim,ar.sim2)

}

hist(simul)

quantile(simul,c(0.0005,0.9995))

0.05% 99.95%

-0.8116713 0.7866148
